# Assessing summer thermal environment in humid cities: Local climate zone perspective

**DOI:** 10.1016/j.isci.2025.112758

**Published:** 2025-05-29

**Authors:** Junjie Wang, Jun Yang, Xiangming Xiao, Yi Bai, Qiyue Zou, Baojie He

**Affiliations:** 1 Human Settlements Research Center, Liaoning Normal University, Dalian 116029, China; 2 School of Humanities and Law, Northeastern University, Shenyang 110169, China; 3 Jangho Architecture College, Northeastern University, Shenyang 110169, China; 4 School of Biological Sciences, University of Oklahoma, Norman, OK 73019, USA; 5 School of Architecture and Urban Planning, Chongqing University, Chongqing 400045, China; 6 Key Laboratory of New Technology for Construction of Cities in Mountain Area, Ministry of Education, Chongqing University, Chongqing 400045, China

**Keywords:** Environmental science, Urban planning

## Abstract

Single land surface temperature (LST) cannot accurately characterize the high temperature and humidity inside cities due to climate warming. Studies on changes in hot and humid urban environments have gradually used near-surface air temperature and humidity. The present study, applying the theory of local climate zones (LCZs), showed that the distribution features of hot humid urban environment and variations across time and space, both at the surface and near-surface level, could facilitate analysis of the evolution of summer heat climate in high-humidity municipalities. According to the results, from 2013 to 2022, the average LST increased by 6.15°C. LCZ8, LCZ10, and compact LCZs were the main warming areas. Relative humidity had a considerable cooling influence on the near-surface temperature but had no discernible effect on LST. Temperature of the wet bulb in the city center was lower than in the suburbs, and those in LCZs 1–3 were higher than those in LCZs 4–6.

## Highlights

• Investigation of the surface and near-surface humid urban thermal environment
• Variations in thermal climate reaction in high humidity areas: an LCZ perspective
• Combining the effects of temperature and humidity indicators
• Extended WRF-UCM model application scenarios

## Graphical abstract

**Figure:**
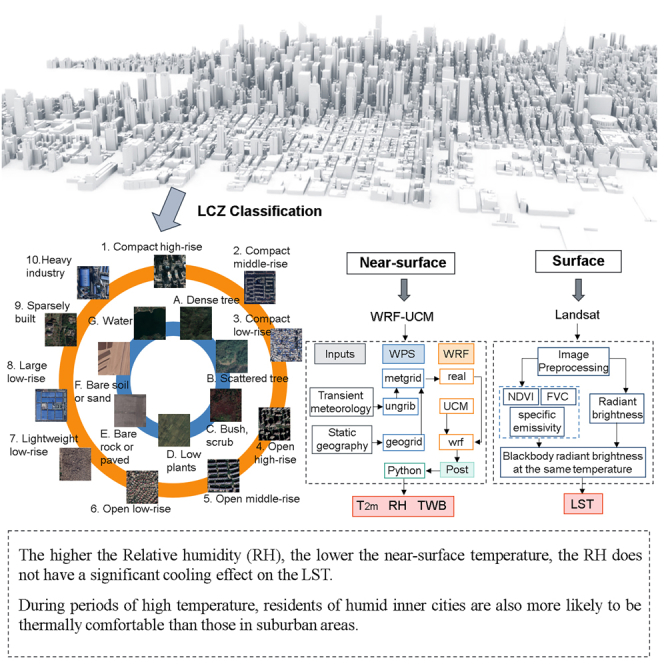

## Introduction

The global temperature has risen by 1.5°C since pre-industrial times, according to the Intergovernmental Panel on Climate Change, and atmospheric water vapor content has increased by 7% per 1°C of warming.^1^^,^^2^ Increasing temperature and humidity makes it increasingly challenging to obtain thermal convenience in warm and humid environments, especially in rapidly developing and densely populated urban areas. The United Nations Sustainable Development Goals explicitly state in Articles 11 and 13 how critical it is to address climate change and establish sustainable cities. Heat islands in urban areas are a common occurrence as urban construction continues to accelerate. In addition to increasing hospitalization and mortality rates for urban residents, which jeopardize their physical and mental health,^3^^,^^4^^,^^5^^,^^6^ the urban heat island (UHI) effect causes urban microclimate, which affects the capacity of a city to develop sustainably.^7^^,^^8^^,^^9^

Heat islands in the surface, urban canopy, and urban boundary layer are all part of the UHI phenomenon.^10^ Scholars have extensively examined the UHI effect and reported that surface heat islands are more susceptible to changes in their outermost characteristics and the actions of humans,^11^^,^^12^^,^^13^ and the intensity is often characterized based on land surface temperature (LST); some scholars have pointed out that 500–650 m is a suitable scale for studying urban temperature for urban characterization.^14^ The variation law of urban near-surface air temperature can be reflected in the distribution characteristics and numerical changes of LST. However, in cities, although the surface temperature can well represent the urban thermal environment at the two-dimensional level, the surface temperature, on its own, cannot adequately represent the effects of the three-dimensional level, such as urban ventilation and air humidity, at the near-surface level.^15^^,^^16^^,^^17^ Near-surface air temperature has a more direct impact on residents’ perceptions of physical health and body surface thermal comfort than surface temperature.^18^^,^^19^ Therefore, near-surface temperature better represents urban dwellers’ comfortable temperatures during work and daily life at the pedestrian level.

Humidity affects the body’s thermoregulation, which in turn affects the body’s perception of temperature and its capacity to adapt. Under similar temperature conditions, higher humidity makes body temperatures significantly higher than the ambient temperature, thus exacerbating the health hazards of high temperatures to residents. The main fields of research on temperature and humidity combinations in humid and hot environments are the psychological comfort index and the comfort variability of hot and humid environments between different climatic zones.^20^^,^^21^^,^^22^ Conversely, majority of current thermal environment research focuses on one aspect, the heat island; less research has been directed at the variability in humid and hot environments, in addition to the spatiotemporal evolution characteristics. Traditionally, meteorological station data are used in the study of hot and humid environments; however, gathering meteorological data is challenging considering the unequal distribution of meteorological stations. Researchers are increasingly using model simulations of observations of urban climates.

The weather research and forecasting (WRF) model and the urban canopy model (UCM) jointly establish the WRF-UCM coupled model, which allows for the simulation of small- and medium-scale meteorological changes in cities. The model has been applied extensively in many cities across the globe, validating the WRF-UCM coupled model’s efficacy as an urban weather/climate simulation system.^23^^,^^24^^,^^25^ Zhu et al.^26^ implemented the WRF-UCM system to explore the effects of urban water bodies on temperature conditions given various building densities from an urban ventilation perspective, and Fedor and Hofierka^27^ explored the effects of changes in urban heat island intensity (UHII) in four common weather scenarios with clear skies, and they reported that air humidity affects UHII significantly. Such studies demonstrate the accuracy and validity of the WRF-UCM model for studying urban thermal environments. In existing studies, most of the simulations using the WRF-UCM model are based on traditional land cover types, and most of the studies on humidity have focused on the radiative effects of water on the periphery; very few scholars have investigated the overall humid-thermal environment of the city in terms of relative humidity (RH) and temperature of wet bulb (TWB).

Rapid urbanization, along with the growth of cities and population explosion, has led to in the replacement of naturally occurring surfaces with man-made surfaces of various kinds.^28^^,^^29^^,^^30^ Human activity and energy exchange processes are closely associated with changes in land use and cover. Surface temperature increases produce numerous negative impacts, including exacerbating heat islands,^31^^,^^32^^,^^33^^,^^34^^,^^35^ and interfering with air, moisture, and energy sources throughout the planet’s crust and the atmosphere, seriously impairing UHI^36^^,^^37^^,^^38^ and influencing regional climate significantly.^39^^,^^40^^,^^41^ According to Chen et al.,^23^ 56.8% of the heat island phenomenon is caused by urban land use. In addition, three-dimensional components, including the sheer count of high-rise apartments and building density, may worsen the negative impacts of UHI by obstructing city-based ventilation and allowing heat to escape.^42^^,^^43^^,^^44^

Stewart and Oke^45^ proposed the local climate zones (LCZs) framework, which divides the city into 17 climatic zones, for the comprehensive exploration of the effects of urban morphology on UHI. Additionally, the LCZ integrates urban spatial morphology into the climate model, emphasizes the city’s three-dimensional morphology, and more effectively integrates the city-climate system. Studies on the city heat climate have benefited considerably from the method, which has transformed urban research.^46^^,^^47^ Zhang et al.^48^ analyzed empirical studies over the past 10 years using the LCZ method through bibliometrics and other techniques, and they confirmed the universality and applicability of the LCZ framework in thermal environment research. To obtain LCZ maps, the LCZ Generator of World Urban Database and Access Portal Tools, remote sensing interpretation, Geographic Information System (GIS) methods, and combined remote sensing-GIS methods are mainly applied.^49^^,^^50^^,^^51^ With advancements in computer technologies, the use of machine learning to identify LCZ types has gradually attracted the attention of scholars.^52^ Among them, the LCZ Generator has been used extensively in research owing to its easy operation, high accuracy, and accessibility. The spatial variability of land use is quantitatively described by the landscape pattern index.^53^^,^^54^ When Liu et al.^55^ made use of the pattern of the landscape index to calculate the LCZ, they found that the index also had a good explanatory line in a three-dimensional spatial pattern. This allowed them to characterize the specific changes in LCZ statistically.t

**Figure 1:**
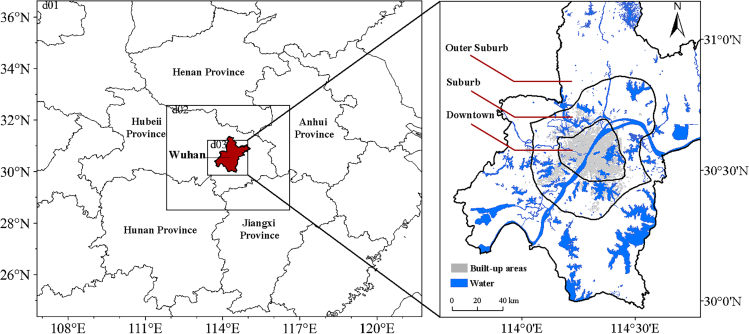
The map illustrating the configuration of the nested layers and an overview of the study area The left side shows the three bi-directional nested layer setups with resolutions of 4.5 km, 1.5 km, and 0.5 km from the outer layer to the inner layer, and the right side shows the actual study area.

The present study uses Wuhan as the study object and uses Landsat8 OLI/TIRS remote sensing data to invert the surface heat island, the WRF-UCM model to simulate the near-surface heat island, RH, and wet-bulb temperature of the city, and meteorological data to correct it (The details of the data are shown in Table 1). This allows the investigation of the summer spatiotemporal variability that defines the humid-thermal climate in Wuhan under the LCZ classification scenarios on a 500-m scale (The scope of the study area is shown in Figure 1). The present study could offer key insights on the temporal and geographical features of urban hygrothermal environment trends at the surface and near-surface levels.

**Table 1:** Data sources

Data	Time	Resolution	Source
Landsat8 OLI/TIRS	2013 and 2022	30 m	https://www.gscloud.cn/
OpenStreetMap	2022	–	https://www.openhistoricalmap.org/
NCEP-FNL	2013 and 2022	1°	https://rda.ucar.edu/datasets/
Meteorological data	2013 and 2022	–	http://data.cma.cn/

## Results

### LCZ classification results

**Figure 2:**
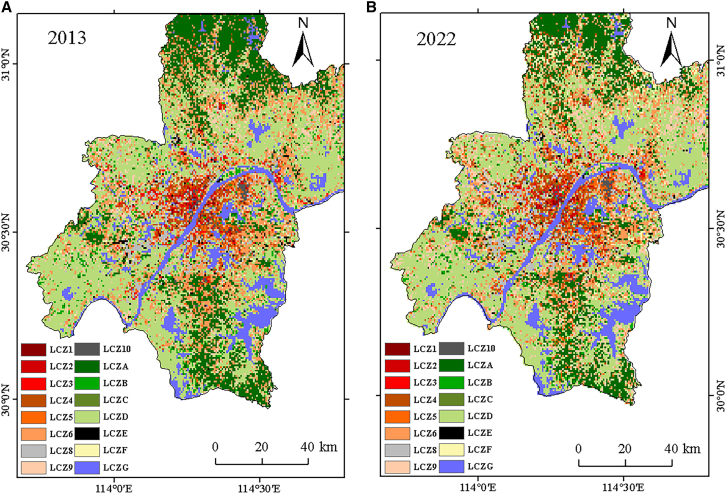
LCZ classification results (A) 2013 results. (B) 2022 results.

The categorization outcomes of the LCZ Generator showed a high classification accuracy of 0.86 for 2013 and 2022 (detailed classification accuracy, as shown in Figure S1). The accuracy of each classification result was high, thus meeting the requirements of the present study. Following resampling of the LCZ results (Figure 2), 24,677 picture components comprised the LCZs of the entire research region. In 2013, the research region’s LCZs of natural types made up 65.66% of the area, while the LCZs of built types made up 34.34%; in 2022, the LCZs of building types accounted for 36.12% of the overall LCZs, and the LCZs of natural types accounted for 63.88%. LCZD is the largest in the study area, distributed in the outer suburbs of the city, mainly farmland paddy field. A large number of LCZGs are distributed in the city, which is a key feature that distinguishes it from other cities. In the area within the third ring road of the city center, LCZ1–5 are mainly concentrated, and in the northeast area of the outer suburbs, LCZ10 is distributed mainly in the towns in the east of the study area. Nature-type LCZs other than LCZGs were mostly dispersed in the Wuhan’s suburban and remote region of the city, among which LCZD pixels had the highest number (8,423 pixels in 2013 and 8,329 pixels in 2022), followed by LCZ6 (4,149 pixels in 2013 and 3,291 pixels in 2022 image elements) and LCZA (3,994 pixels in 2013 and 3,291 pixels in 2022). The largest change between 2013 and 2022 was in LCZ9, with an increase of 896 pixels, followed by LCZ6, with a decrease of 858 pixels, and LCZA (a decrease of 703 pixels).

**Figure 3:**
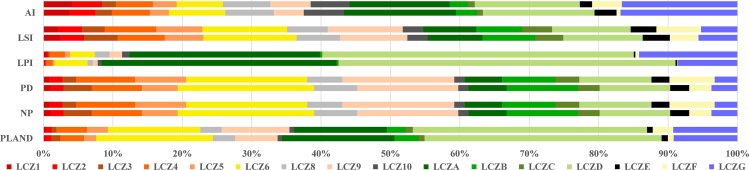
LCZ division results 2013 results (top) and 2022 results (bottom).

Based on the landscape pattern delineation approach (Table 2), the LCZ was examined from three perspectives, including the quantity, the form, and the degree of patch aggregation, to better characterize the changing law of the LCZ (Figure 3). As shown in Table S4, the PLAND of LCZ9 changed the most in the study area, with the highest degree of growth of 3.63%, followed by LCZ6, which shrank by 3.49%. NP and patch density (PD) reflected the changes in the number of patches in LCZs. LCZ6 had the largest values (NP was 1,130 in 2013 and 1,096 in 2022, and PD was 1,833,002,149.32/hm^2^ and 1,776,553,065.61/hm^2^, respectively), and LCZF had the most change. NP increased by 226 and PD increased by 366,115,310.59/hm^2^. LCZD was the most dominant patch type and had the most change, with a decrease of 4.83% in LPI between 2013 and 2022. Landscape shape index (LSI) reflects the complexity of the shape of LCZ patches, with LCZF patches showing the greatest change in shape complexity, increasing by 8.78% between 2013 and 2022. The largest aggregation index (AI) share was taken by LCZG (61.03% in 2013 and 62.23% in 2022), followed by LCZA (58.40% in 2013 and 53.78% in 2022) and LCZD (58.28% in 2013 and 56.59% in 2022). The largest change was in LCZF (13.92% increase), which not only increased in number but also in connectivity and agglomeration.

**Table 2:** Landscape pattern indexes and their ecological significance

Index	Unit	Formula	Ecological significance
PLAND	%	$PLAND=\sum_{j=1}^{n}a_{ij}/A\times 100\%$	the percentage of patch types in the landscape
NP	number	$NP=\sum_{j=1}^{n}N_{i}$	count of patches
PD	number/hm^2^	PD=NP/A	count of patches for each area unit
LPI	%	$LPI=\mathrm{Max}\left(a_{1},a_{2},\ldots ,a_{n}\right)/A\times 100\%$	the proportion of the largest patch in the entire landscape
LSI	–	$LSI=0.25E/\sqrt{A}$	plaque shape complexity
AI	%	$AI=\left[g_{ii}/\max⟶g_{ii}\right]\times 100\%$	interconnectivity among each sort of landscape patches

Where A is the total landscape area, and a_ij_ is the area of patch ij. N_i_ is the count of class i plaques, and E is the total perimeter of the plaque. The count of equivalent adjacent to patches in the essential landscape type is called g_ii_.

### LST spatio-temporal variations and relationship with LCZs

**Figure 4:**
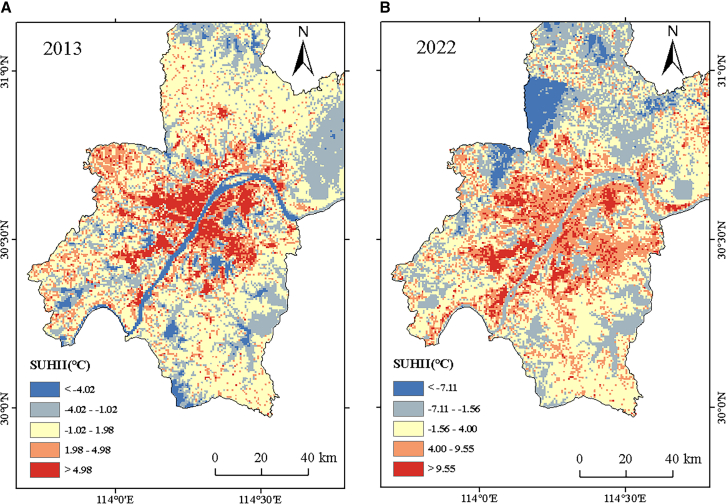
SUHII calculation result (A) 2013 results. (B) 2022 results. SUHII, surface heat island intensity.

To ensure that the investigation was conducted correctly, the estimated results were resampled to 500 m. The surface temperature was divided into five grades using the mean and standard deviation method. The specific classification is shown in Table 3. The thermal environment’s surface temperature class distribution in the investigation area is depicted in Figure 4. In the research subject matter, the average LST at the LCZD was 30.13°C in 2013 and 36.28°C in 2022. Most of the high- and sub-high-temperature areas were covered by roadway, Wuhan City Bypass Highway, with the city center having the largest concentration. In 2013 and 2022, the heat island areas made up 25.25% and 28.39% of the study area, respectively. Wuhan’s surface high-temperature zones and sub-high-temperature zones expanded its urban core to its outskirts by 0.39 km^2^. The expansion was positively and strongly connected with the trend of the LCZs of the building type. Water features, including rivers and lakes, were the primary cold island regions in the research domain. Zones with low and inferior temperatures have been defined as cold island regions. The study region is dotted with big and small lakes. The Yangtze River flows through the heart of Wuhan, and green spaces are mostly found in the farther-flung suburbs.

**Table 3:** LST classification level table

LST level	Grading method	2013 value range	2022 value range
Low temperature area	T ≤ A-1.5 SD	≤ −4.02°C	≤ −7.11°C
Sub-low temperature area	A-1.5 SD < T ≤ A-0.5 SD	−4.02°C to −1.02°C	−7.11°C to −1.56°C
Medium temperature area	A-0.5 SD < T ≤ A+0.5 SD	−1.02°C to 1.98°C	−1.56°C to 4.00°C
Sub-high temperature area	A+0.5 SD < T ≤ A+1.5 SD	1.98°C–4.98°C	4.00°C–9.55°C
High temperature area	T > A+1.5 SD	>4.98°C	>9.55°C

T is the UHII value, A is the average (in 2013, A = 0.48°C, and by 2022, A = 1.22°C), where SD signifies the deviation from the mean (in 2013, SD = 3.00°C and in 2022, SD = 5.55°C).

**Figure 5:**
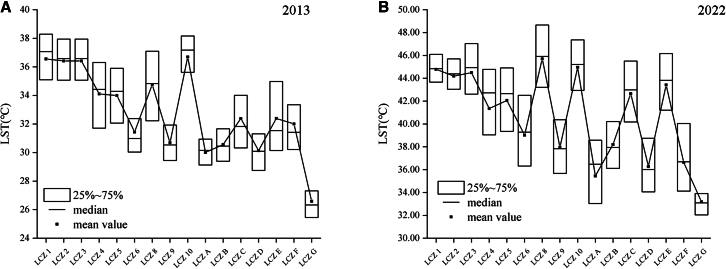
Boxplot of LCZ vs. LST (A) 2013 results.(data are represented as mean ± SEM). (B) 2022 results (data are represented as mean ± SEM).

Figure 5 shows that between 2013 and 2022, the study area’s average summer surface temperature rose dramatically. In terms of trend, the total LST of LCZs of the natural type was lower than that of LCZs of the building type, and within the building type, the LST of LCZs of the open type was much lower than LST of LCZs of the compact type. Among LCZA-G, LCZC (average 32.39°C in 2013 and 42.66°C in 2022) and LCZE (flat 2013 32.39°C and average 43.42°C in 2022) had the highest LST, while LCZG (2013 temperatures were 26.59°C, whereas in 2022, they were 33.19°C) had the lowest. The study area has a dense water network and dense vegetation in the far outskirts of the city, which is the main cooling area within the city. In building type LCZs, the LST of compact LCZs was often higher than that of open LCZs; while among the compact LCZs, LCZ1, with its higher floor heights that have the effect of shading the solar radiation, did not have significantly higher LST than the other compact LCZs. LCZ8 and LCZ10 had significantly higher LST than the other low-rise LCZs due to the relative clustering of their spatial distribution and the use of building materials. LCZ8 and 10 are the main warming zones of the city along with the compact LCZs in the 10-year evolution.

### WRF-UCM simulation results and relation to LCZ

**Figure 6:**
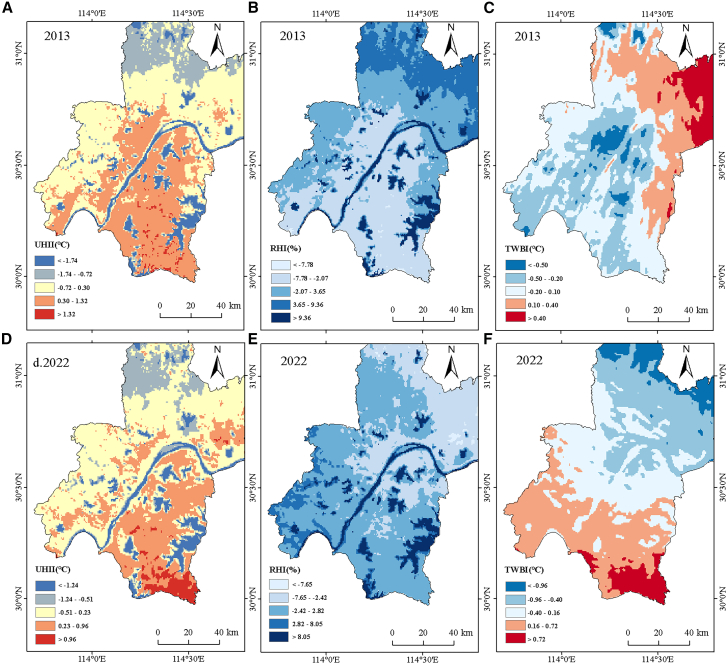
Class distribution of WRF-UCM simulation results (A–F) Class distribution of WRF-UCM simulation results (A and D are UHII results for air temperature simulation at 2 m; B and E are RHI results for RH intensity simulation; and C and F are TWBI results for wet bulb temperature simulation). (A, B, and C) are 2013 results and (D, E, and F) are 2022 results.

To describe the WRF-UCM simulation results better, the processed data were classified into gradients using the method of average deviation standard deviation (Figure 6). However, the central area with dense buildings inside the city shows a relatively stable and independent state at 500-m resolution.

**Figure 7:**
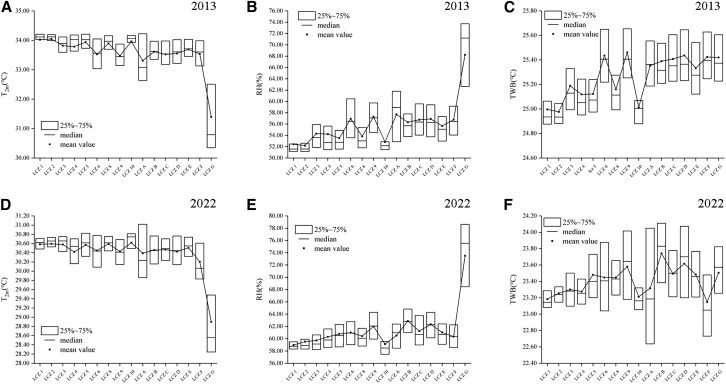
Boxplots of T2m, RH, and TWB versus LCZ (A, B, and C) 2013 results (data are represented as mean ± SEM). (D, E, and F) 2022 results (data are represented as mean ± SEM).

Near-surface heat islands were concentrated in the urban center, that is in the building type LCZs (mean temperature 33.56°C in 2013 and 30.43°C in 2022); evidently, near-surface temperatures near water bodies were lower than in the surrounding areas, and the area of cooling radiation from the water bodies varied according to wind speed and direction. The LCZA was mainly distributed in the region to the north, and the patches were aggregated, which resulted in a better cooling effect. The amount of LCZA in the northern region declined in 2022 compared to in 2013, and the degree of patch fragmentation rose. In the southern part of the study area, the near surface temperature in 2013 and 2022 was higher than that in other areas, especially in 2022 The area had the highest near surface temperature. This is due to the existence of continuous mountains in the south of Xianning City outside the study area, where accumulated heat is deposited under the influence of prevailing winds. The cooling effect of LCZG was most pronounced, as Figure 7 shows, and building-type LCZs frequently had near-surface air temperatures greater compared to LCZs of the natural type. Temperature values were greater in high-rise LCZs than in low-rise LCZs of the same kind, and within LCZs of the building type, compact LCZs had generally higher air temperatures than open LCZs.

Overall RH was high in the study area, with averages of 56.90% in 2013 and 62.32% in 2022. Temperature decreased with increasing humidity, with the mean temperature in 2022 being 3.13°C lower than the mean temperature in 2013; the RH, however, had less effect on LST, with the overall LST in 2022 being higher than that in 2013. In terms of spatial distribution, the city center consistently showed lower RH, with higher RH in densely vegetated areas, such as the far suburbs. The highest RH was concentrated in water bodies, especially in the city center, which had an obvious “humidifying” effect on the surrounding areas, alleviating the dryness of the surrounding areas. Combined with the LCZ perspective (Figure 7), compared to the built-type LCZs, the natural-type LCZs had greater total RH, and the highest RH was observed in the LCZG. Within the built-type LCZs, compact LCZs were smaller than open-type LCZs, and vegetated LCZs were larger than non-vegetated LCZs.

To better explore the integrated changes in humid and thermal environments at the habitat level, the TWB was added to the present study. The average TWBs in the research region were 25.44°C in 2013 and 23.62°C in 2022. The TWB values were lower in areas within the peri-urban area and concentrated and more stable in the city center, which was much less affected by the wind environment than the suburban and far suburban areas. TWB was lower for compact LCZs than for open LCZs and lower for high-rise LCZs than for low-rise LCZs at the same density among building-type LCZs; for LCZs of the same height, the denser LCZ correlated with lower TWB (26.58°C). Among the nature-type LCZs, there were fewer LCZAs and more fragmented landscapes in 2022; thus, the distribution of TWB values in 2022 was more dispersed across the LCZA classifications.

## Discussion

### Model accuracy validation

**Figure 8:**
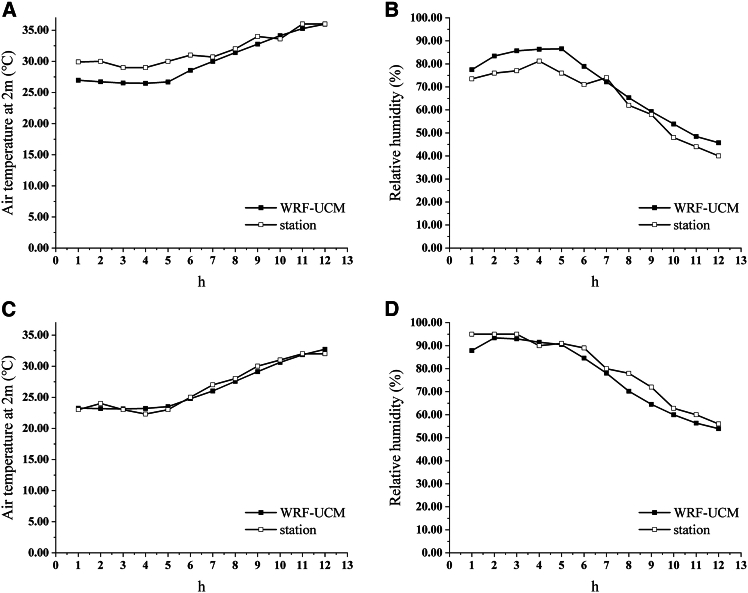
Comparison of WRF-UCM simulation results with measured data at meteorological sites (A and B) 2013 results. (C and D) 2022 results.

Prior research on urban near-surface environments have used ERA5, NOAA reanalysis, and Moderate Resolution Imaging Spectroradiometer (MODIS) data, which are more accessible but have lower temporal and spatial resolutions and are not suitable for small areas and complex inner cities.^56^^,^^57^ High-density automated station data, although with higher data accuracy, are more difficult to obtain and can only be obtained for a particular point where the station is located, which does not accurately describe continuous changes in the entire space.^58^^,^^59^ Figure 8 illustrates a comparison between the air temperature and RH data from WRF-UCM simulation results computed in this work and the measured data from the meteorological stations. The results demonstrate strong similarity between the simulation results and the observations from the meteorological stations. As a result, the UCM based WRF simulation setup is accurate, and the simulation results are dependable and can be utilized in this study in place of meteorological station data, which is unable to be used due to missing and uneven distribution, to give a more precise account of the urban hygrothermal environment.

### Spatiotemporal characteristics of the thermal environment in humid cities

#### Near-surface air temperature and LST characteristics under wind speed and direction

**Figure 9:**
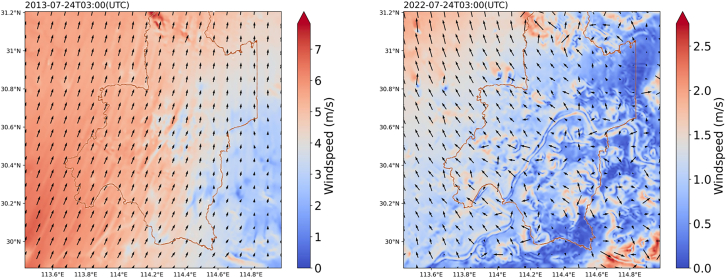
WRF-UCM wind speed and direction simulation result 2013 results (left) and 2022 results (right).

Little research has been conducted at the near-surface level, and most earlier investigations of the urban thermal environment were focused on variations in LST. The study included a near-surface air temperature indicator. The comparison’s findings demonstrated that, on average, building-type LCZs was more extreme compared to that of natural-type LCZs; however, the LST response to LCZ was more sensitive and the numerical change in LST was greater than that of the near-surface temperature. At the 500-m resolution, air temperature’s response to wind direction and speed (Figure 9) was notable, whereas wind speed and direction had less effect on LST. Changes in wind direction affect the direction of heat radiation, and wind speed affects the degree and speed of heat dissipation, which is significant for ambient heat accumulation.^60^^,^^61^ Combining Figures 7A, 7B, and 9, in the low wind speed region, the heat dissipation rate is slower, which leads to heat accumulation in the city. Furthermore, the radiation effect of the cooling areas, such as plant and water bodies, to the surrounding features will be weakened. In complex cities, urban ventilation is blocked because of the obstruction of buildings, and there is no good ventilation corridor inside the city to carry away the heat generated by solar radiation and human activities within a certain range of wind direction changes. Thus, the impact of urban building resistance on heat transmission in the direction of the dominant wind can be taken into account in the assessment of the city’s the heat environment,^62^^,^^63^ and according to the most prevalent wind’s direction, we can build ventilation corridors in the city,^64^ to better use the natural breeze to alleviate the city’s high temperature issue.

#### Effect of humidity on near-surface air temperature and LST

Relatively few studies have combined temperature and humidity; most research^65^^,^^66^ has concentrated on the separate changes in dry and wet islands. In the present study, we examined RH and found that it has a non-significant cooling influence upon the LST but a significant freezing contribution to the near-surface air temperatures. RH is closely linked to thermal comfort.^67^^,^^68^ Both the land cover and the overall climate in the study area changed between 2013 and 2022. Both LST and RH in the summer of 2022 are higher than those in 2013, but the near-surface air temperature in 2022 was lower than that in 2013 under the influence of humidity. Over time, the city has undergone tremendous change, so that the study of urban environment should enhance the real-time. At the surface level, the LST of the surrounding areas is not cooled considerably by LCZG; however, at the near-surface level, it is cooled and humidified considerably. Conversely, it has more substantial freezing and moisturizing effects at the near-surface level, where higher wind speeds can transfer moisture from the LCZG to a broader area in the peripheral area, and it barely cools LST in the periphery area (Figure 9). Within a general city, there is relatively little surface vegetation and natural water bodies, and the higher building heights are more densely distributed, which hinders urban ventilation and reduces water vapor flow. According to Figure 9, the humidification effect of waterways and woods distributed in the periphery of the city cannot be transmitted to dense built-up areas within the city.

Inside a humid city, TWB, which combines RH and air temperature, is more integrated to reflect differences in outdoor thermal comfort at the level of residents’ body sensations.^69^^,^^70^ The study’s findings indicate that the TWB and air temperature in URA vary. The highest temperatures were found in urban centers, where lower humidity results in lower TWB than in the surrounding suburbs. The shadowing impact caused by high-rise buildings, as well as the obstruction of airflow in high-humidity cities, can make it easier for residents inside the area to obtain thermal comfort compared with open LCZs. Based on a comparison between 2013 and 2022, the built-up area inside the city has a relatively stable structure, and the TWB remains in a relatively stable range. Therefore, when exploring urban thermal environments in future studies, humidity indicators should be added selectively to urban wet and dry conditions.

### Conclusions

Based on the LCZ framework, the present study reveals temporal and spatial variations and distribution characteristics of urban humid and thermal environment from surface and near-surface levels. The majority of high- and sub-high-temperature areas were mostly distributed within the metropolitan center. The research region’s average summer LST increased by 6.15°C as a result of building type LCZ growth, and the total count of high- and sub-high-temperature regions increased by 0.39 km^2^. Furthermore, compared to the open LCZs, the LSTs of the compact LCZs were often greater. The two main temperature-increasing zones in the city were the small LCZs and LCZs 8 and 10.

The results of the WRF-UCM simulation show that in the overall change of the city in 2022 and 2013, the higher the RH, the lower the temperature. While the near-surface temperature had been cooled drastically by the RH, the LST was not particularly cooled by it. Due to a decline in LCZAs and an increase in fragmentation, the cooling impact was lessened at the urban near-surface level. This suggests that the distribution of the built-up region was lower than the open space and the urban center was lower than the periphery suburbs. With the same degree of densification, there were fewer high-rise LCZs than low-rise LCZs, and denser LCZs were correlated with lower TWB among LCZs of the same height. Among the LCZs of the building type, LCZs 1–3 had a higher TWB than LCZs 4–6.

This study confirmed that in humid areas, a single LST index is not very accurate in representing the thermal comfort of residents’ production and life, and the cooling effect of RH on the near-surface may lead to higher LST but a relatively lower near-surface air temperature. Therefore, in hot summer, the high temperature inside the city in drier areas can be reduced by increasing the air humidity, for example, by spraying water at noon, building artificial lakes, and other measures, to alleviate the disasters caused by high temperature. In the same humid city, built-up areas with higher building density in the center are drier than suburban areas with lower building density, where it may be easier to obtain thermal comfort. Therefore, more sound early warning measures should be established for residents in suburban areas, especially in agricultural farming areas, to reduce the impact of high humidity and high temperature.

### Limitations of the study

The present study had some limitation, requiring additional investigations. First, this research describes the urban spatial morphology and changes in the hygrothermal environment in Wuhan during urbanization; however, studies on long time series or diurnal scales need to be improved. Second, cloudiness also affects RH and LST, and the present study only considered changes in the urban hygrothermal environment under clear and cloudless weather, while LST, near-surface air temperature, and RH may show different patterns of change in cloudy weather.

## Resource availability

### Lead contact

Further information and requests for resources should be directed to and will be fulfilled by the lead contact, Jun Yang (yangjun8@mail.neu.edu.cn).

### Materials availability

This study did not generate new unique materials.

### Data and code availability

• All statistical data reported in this paper will be shared by the lead contact upon request.
• This paper does not report the original code.
• Any additional information required to reanalyze the data reported in this paper is available from the lead contact upon request.

## Acknowledgments

This research study was supported by 10.13039/501100018617Liaoning Revitalization Talents Program (grant no. XLYC2202024), Basic Scientific Research Project (Key Project) of the Education Department of Liaoning Province (grant no. LJKZ0964), the 10.13039/100020593Fundamental Research Funds for the Central Universities (grant no. N2111003), 10.13039/501100005329Natural Science Foundation of Guizhou Province (grant no. [2019]1150).

This manuscript has not been published or presented elsewhere in part or in entirety and is not under consideration by another journal. We have read and understood your journal’s policies, and we believe that neither the manuscript nor the study violates any of these.

## Author contributions

J.-J.W., conceptualization, validation, formal analysis, and writing – original draft; J.Y., conceptualization, methodology, and review; X.-M.X., visualization and methodology; Y.B., writing – original draft and methodology; Q.-Y.Z., writing – original draft; B.-J.H., conceptualization and methodology.

## Declaration of interests

The authors declare no competing interests.

## STAR★Methods

### Key resources table

**Table:** 

REAGENT or RESOURCE	SOURCE	IDENTIFIER
**Deposited data**		

Landsat8 OLI/TIRS	US Geological Survey (USGS)	earthexplorer.usgs.gov/
Road data	Open street map	www.openstreetmap.org/
NCEP-FNL	National Center for Atmospheric Research (NCAR)	rda.ucar.edu/datasets/
LCZ maps	Demuzere et al.^71^^,^^72^	zenodo.org/records/6364594

**Software and algorithms**		

ArcGIS for Desktop Basic	ESRI	www.arcgis.com; RRID:SCR_011081
ENVI 5.3	ESRI	envi.geoscene.cn
WRF 4.5	National Center for Atmospheric Research (NCAR)	www.mmm.ucar.edu/models/

### Experimental model and study participant details

This study did not involve experimental models or study participants.

### Method details

#### Study area

Wuhan, with its 8569.15 km^2^ of land and 166 lakes, is a city in central China. It is a key hub for the strategy of central China’s ascent and one of the principal cities of the Yangtze River’s mid-section metropolitan cluster. Wuhan is referred to as a “hot stove” because of its subtropical monsoon climate, which features high temperatures, rainy summers, 34°C on average throughout the year, and up to 80% average humidity. In 2013, Wuhan experienced a heat disaster with 155 heatstroke victims and five deaths, which was the first-time deaths were reportedly caused by high temperature. The year 2022 saw 33 days of high temperatures in Wuhan, with daily maximum temperatures exceeding 40°C in some areas. Wuhan was chosen as the research location because human health and the sustainable city growth are tied to hot and humid environments. An overview of the research area is shown in Figure 1.

#### Data

Using Landsat data, the LST was inverted for the current investigation. The city center, suburban, and peri-urban areas of Wuhan were delineated using road network data, and the initial field data for the WRF-UCM simulation were obtained from meteorological initialization field data and LCZ data, with meteorological station data used to assess the relevancy of the WRF-UCM model inversion. The LCZ data were obtained using the LCZ generator of the WUDAPT platform (http://lcz-generator.rub.de) tool on the WUDAPT platform. Due to the limited return period of Landsat data and the interference of cloudiness, data from July 24, 2013 and 2022 were chosen in the current investigation to represent the LST of Wuhan in summer (collected at roughly 3:00 GMT). To ensure data consistency, the WRF-UCM was used to simulate the data simultaneously. The details of the data are presented in Table 1.

#### LCZ division

The LCZ Generator, an online web application platform for creating LCZ maps proposed by Demuzere et al.,^71^^,^^72^ integrates state-of-the-art LCZ mapping while providing automatic accuracy assessments, training data derivatives, and new methods for identifying suspected training zones, thereby greatly simplifying the workflow and enhancing the accessibility and availability of LCZ maps, thus facilitating the dissemination of LCZ concepts and maps. There are three primary phases involved in creating LCZ maps with the LCZ Generator. First, Google Earth Pro is used to select training samples. Second, the training zone information is uploaded to a web application. Ultimately, the mailbox downloads the accuracy evaluation report and the classification results to create an LCZ image with a 30 × 30-m resolution. Employing the nearest probable neighbor method, the entire research region was segmented into 824167 pixels and reconfigured to an approximate 500 m. We evaluated the accuracy of LCZ images after resampling and constructed a confusion matrix. The results indicated that (Tables S1 and S2) the overall accuracy of resampling in 2013 was 88.30%, with a Kappa coefficient of 85.36%. In 2022, the overall accuracy increased to 91.00%, and the Kappa coefficient rose to 89.05%.

The Wuhan City Master Plan designated the area encompassed by the Wuhan Bypass Highway as the town’s outer suburbs and everything inside the third ring road as the Wuhan core region. Owing to the large urban area of Wuhan, the northern part of the study area could not be obtained with the same classification result when using WUDAPT to classify LCZ types. Therefore, to ensure the consistency of the LCZ classification accuracy within the region, Wuhan City was cut in accordance with the findings of the LCZ categorization; Table S3 lists the various LCZ kinds.

To better explore the changing law of the LCZ, the obtained LCZ classification results were divided from the perspective of landscape patterns using FRAGSTATS 4.5 (https://fragstats.org/). Six indices were selected: landscape type proportion (PLAND), count of patches (NP), patch density (PD), maximum patch index (LPI), landscape shape index (LSI) and aggregation index (AI) (Table 2).

#### Land surface temperature retrieval

Landsat8 OLI/TIRS data were utilized in this investigation. The absolute calibration accuracy of TIRS channel 11 had a large error, and band 10 was the best band for inverting the surface temperature. In addition, Qin et al.^73^’s single-window technique has been used extensively and has high accuracy; therefore, it was selected as the LST inversion model in the present study. Its equation is as follows:

(Equation 1) $$\begin{array}{c} T_{S}=\frac{\left\{a\left(1-C-D\right)+\left[b\left(1-C-D\right)+C+D\right]\times T_{10}-DT_{a}\right\}}{C} \end{array}$$

Where $T_{S}$ is the value of the true surface temperature (°C); *a*, *b* are constants, a = -67.355351 and b = 0.458606; the brightness temp that applies to TIRS’s 10th band is $T_{10}$; $T_{a}$ is the average climatic action degree; while C, D are the intermediate factors established from the airborne reception τ and the surface specific radiance ε using the equation below:

(Equation 2) $$\begin{array}{c} C=\tau \varepsilon \end{array}$$

(Equation 3) $$\begin{array}{c} D=\left(1-\tau \right)\left[1+\left(1-\varepsilon \right)\tau \right] \end{array}$$

The urban-rural dichotomy is the basis for the majority of conventional UHI estimates. However, this approach has certain shortcomings with regard to defining urban and rural district (URA). Consequently, the delineation method proposed by Stewart and Oke,^45^ which characterizes the UHII by calculating the temperature variation between various LCZ types and a particular natural LCZ type, LCZD (low plants), was employed in this investigation to enhance comprehension of the connection between heat island impacts and LCZ types. The equation used is as follows:

(Equation 4) $$\begin{array}{c} {SUHII}_{LCZX}=T_{LCZX}-T_{LCZD} \end{array}$$

where ${SUHII}_{LCZX}$ is the SUHII of LCZX (°C), $T_{LCZX}$ is the current temperature of LCZX (°C) and $T_{LCZD}$ is the mean temperature of LCZD (°C). Using the standard deviation of the mean approach, the data were categorized into five classes (Table 3).

#### WRF nested domain and boundary condition settings

The fifth-generation mesoscale model, WRF is a mathematical forecasting system at the mesoscale scale that was built using MM4. WRF version 4.5 was the mathematical system employed in this investigation, which incorporates an UCM with an integrated single-layer city. The features of urban topography serve as the basis for the UCM, which can more accurately respond to the characteristics of physical processes, such as land types, multiple longwave and shortwave reflective processes between buildings and the ground, and between buildings and the sky in the city, and can more accurately describe the changes within a complex city. The horizontal domain used in this experiment consisted of 301 × 301 (4.5 km), 301 × 301 (1.5 km), and 301 × 301 (0.5 km) raster points in three bidirectional nested domains. The center point’s latitude and longitude were 30.59° E and 114.27° N, respectively. The smallest domain (D03) covered almost all of Wuhan. The simulations were performed using the UCM standard heat capacity, conductivity, albedo, emissivity, roughness, heat, and momentum of the roof, road, and wall surfaces.^74^^,^^75^ The initial and lateral boundary conditions were obtained from the National Centers for Environmental Prediction (NCEP) Global Prediction System final analysis dataset (with a horizontal resolution of 1 ° × 1 °) with a return period of every 6 h. The LCZ maps provided the basic land-cover and land-use information necessary to conduct simulations.

Three parameters, near-surface temperature (T_2m_), relative humidity (RH), and wet-bulb temperature (TWB), were selected for this study. To better describe the relationship between the simulation results and the LCZ model, the above three parameters were classified and calculated using the method of Stewart and Oke^45^ (see Equations 5, 6, and 7), and the standard deviation of the average approach was used to separate the data into five classes.

(Equation 5) $$\begin{array}{c} {UHII}_{LCZX}=T_{LCZX}-T_{LCZD} \end{array}$$

(Equation 6) $$\begin{array}{c} {RHI}_{LCZX}={RH}_{LCZX}-{RH}_{LCZD} \end{array}$$

(Equation 7) $$\begin{array}{c} {TWBI}_{LCZX}={\text{TWB}}_{LCZX}-{TWB}_{LCZD} \end{array}$$

where $T_{LCZX}$ is the near-surface air temperature of LCZX (°C), $T_{LCZD}$ is the mean temperature of LCZD (°C), and ${UHII}_{LCZX}$ is the UHII of LCZX (°C). The relative humidity intensity (%) of LCZX is represented by ${RHI}_{LCZX}$, the relative humidity (%) by ${RH}_{LCZX}$, and the mean relative humidity (°C) by ${RH}_{LCZD}$. The TWBI (°C) of LCZX is represented by ${TWBI}_{LCZX}$, the TWB (°C) by ${TWB}_{LCZX}$, and the mean TWB (°C) by ${TWB}_{LCZD}$ (Figure 6).
