# Supplementary material for: Assessing summer thermal environment in humid cities: Local climate zone perspective
Source: iScience. 2025 May 29;28(7):112758. doi: 10.1016/j.isci.2025.112758 (PMC12205627; doi:10.1016/j.isci.2025.112758)
Supplement: Document S1. Figure S1 and Tables S1–S4 [file mmc1.pdf]

iScience, Volume *28*

Supplemental information

Assessing summer thermal environment in humid cities: Local climate zone perspective

Junjie Wang, Jun Yang, Xiangming Xiao, Yi Bai, Qiyue Zou, and Baojie He

Supplementary Information for

Assessment of the summer thermal environment in humid cities using WRF-UCM: A local climate zone perspective

This Supplementary Information includes:

1. Supplementary Figure: Fig. S1
2. Supplementary Tables: Table S1, Table S2, Table S3 and Table S4

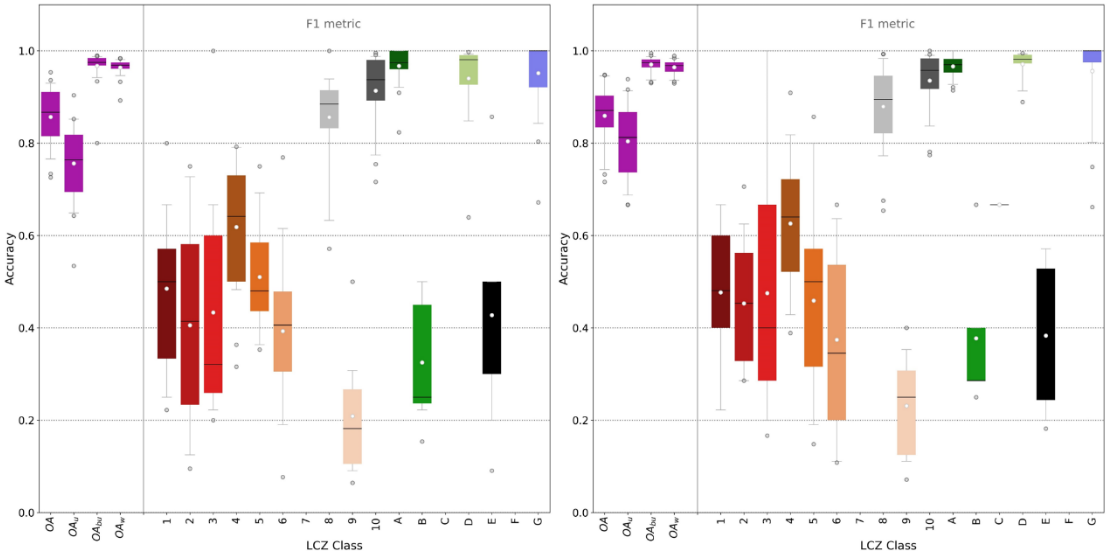

Fig. S1 Schematic of LCZ classification accuracy (2013 on the left, 2022 on the right, related to Figure 2)

Table S1 LCZ confusion Matrix results for 2013

	**LCZ 1**	**LCZ 2**	**LCZ 3**	**LCZ 4**	**LCZ 5**	**LCZ 6**	**LCZ 8**	**LCZ 9**	**LCZ 10**	**LCZ A**	**LCZ B**	**LCZ C**	**LCZ D**	**LCZ E**	**LCZ F**	**LCZ G**	**Total**	**U_Accuracy**	**Kappa**
**LCZ1**	**2**	0	0	1	0	0	0	0	0	0	0	0	0	0	0	0	3	66.67%	
**LCZ2**	0	**1**	0	0	0	0	0	0	0	0	0	0	0	0	0	0	1	100.00%	
**LCZ3**	1	0	**7**	0	0	0	1	0	0	0	0	0	0	0	0	0	9	77.78%	
**LCZ4**	0	0	1	**33**	1	2	1	0	1	0	1	0	2	0	0	0	42	78.57%	
**LCZ5**	0	0	1	0	**11**	1	0	0	0	1	0	0	1	0	0	0	15	73.33%	
**LCZ6**	0	0	1	3	3	**152**	3	2	0	2	1	0	2	0	2	1	172	88.37%	
**LCZ8**	0	0	0	2	1	0	**28**	0	1	0	1	0	2	1	0	0	36	77.78%	
**LCZ9**	0	0	1	0	0	4	0	**40**	0	1	3	0	2	0	0	3	54	74.07%	
**LCZ10**	0	0	1	0	0	0	1	0	**6**	0	0	0	0	0	0	0	8	75.00%	
**LCZA**	0	0	0	1	1	2	0	1	0	**141**	1	1	8	0	0	1	157	89.81%	
**LCZB**	0	0	0	0	0	1	0	2	0	0	**26**	0	6	0	0	0	35	74.29%	
**LCZC**	0	0	0	0	0	1	1	0	0	0	0	**5**	0	0	0	0	7	71.43%	
**LCZD**	0	0	1	1	1	2	0	2	0	2	1	0	**344**	2	1	4	361	95.29%	
**LCZE**	0	0	0	0	0	1	0	0	0	1	0	0	1	**8**	0	0	11	72.73%	
**LCZF**	0	0	0	0	1	0	0	0	0	0	0	0	1	0	**7**	0	9	77.78%	
**LCZG**	0	0	0	1	0	1	0	1	0	2	0	0	3	0	0	**72**	80	90.00%	
**Total**	3	1	13	42	19	167	35	48	8	150	34	6	372	11	10	81	1000		
**P_Accuracy**	66.67%	100.00%	53.85%	78.57%	57.89%	91.02%	80.00%	83.33%	75.00%	94.00%	76.47%	83.33%	92.47%	72.73%	70.00%	88.89%		**88.30%**	
**Kappa**																			**85.36%**

Table S2 LCZ confusion Matrix results for 2022

	**LCZ 1**	**LCZ 2**	**LCZ 3**	**LCZ 4**	**LCZ 5**	**LCZ 6**	**LCZ 8**	**LCZ 9**	**LCZ 10**	**LCZ A**	**LCZ B**	**LCZ C**	**LCZ D**	**LCZ E**	**LCZ F**	**LCZ G**	**Total**	**U_Accuracy**	**Kappa**
**LCZ1**	**1**	0	0	1	0	0	0	0	0	0	0	0	0	0	0	0	2	50.00%	
**LCZ2**	1	**9**	0	0	1	0	0	0	0	0	1	0	0	0	0	0	12	75.00%	
**LCZ3**	0	0	**3**	0	1	0	0	0	0	0	0	0	1	0	0	0	5	60.00%	
**LCZ4**	0	0	1	**36**	1	1	0	0	1	0	0	0	0	1	1	0	42	85.71%	
**LCZ5**	0	1	0	2	**26**	0	1	2	1	0	0	1	0	0	0	0	34	76.47%	
**LCZ6**	0	1	0	1	1	**114**	1	1	0	1	0	1	1	0	0	0	122	93.44%	
**LCZ8**	0	0	0	1	0	0	**14**	1	0	0	0	0	1	0	0	0	17	82.35%	
**LCZ9**	0	0	0	1	1	3	1	**96**	0	1	1	1	2	0	0	1	108	88.89%	
**LCZ10**	0	0	0	0	0	0	1	0	**5**	0	0	0	0	0	0	0	6	83.33%	
**LCZA**	0	0	0	1	1	2	0	2	0	**118**	3	1	1	0	1	0	130	90.77%	
**LCZB**	0	1	0	0	0	1	0	1	0	1	**18**	0	1	0	0	0	23	78.26%	
**LCZC**	0	0	0	0	1	1	0	0	0	0	0	**9**	1	0	0	0	12	75.00%	
**LCZD**	0	0	1	1	1	2	1	3	0	2	2	1	**334**	1	1	0	350	95.43%	
**LCZE**	0	0	0	0	0	1	0	0	0	0	0	0	1	**7**	1	0	10	70.00%	
**LCZF**	0	0	0	0	0	0	1	0	0	1	0	0	1	1	**29**	0	33	87.88%	
**LCZG**	0	0	0	0	0	2	0	0	0	0	0	0	1	0	0	**91**	94	96.81%	
**Total**	2	12	5	44	34	127	20	106	7	124	25	14	345	10	33	92	1000		
**P_Accuracy**	50.00%	75.00%	60.00%	81.82%	76.47%	89.76%	70.00%	90.57%	71.43%	95.16%	72.00%	64.29%	96.81%	70.00%	87.88%	98.91%		**91.00%**	
**Kappa**																			**89.05%**

Table S3 Names and designations of the LCZ types

Building LCZs	Explanation	Nature LCZs	Explanation
LCZ 1	Compact high-rise	LCZ A	Dense trees
LCZ 2	Compact mid-rise	LCZ B	Scattered trees
LCZ 3	Compact low-rise	LCZ C	Bush, scrub
LCZ 4	Open high-rise	LCZ D	Low plants
LCZ 5	Open mid-rise	LCZ E	Bare rock or paved
LCZ 6	Open low-rise	LCZ F	Bare soil or sand
LCZ 7	Lightweight low-rise	LCZ G	Water
LCZ 8	Large low-rise		
LCZ 9	Sparse low-rise		
LCZ 10	Heavy industry		

Table S4 Calculated results for each landscape index of LCZ related to Figure 3

Types	PLAND		NP		PD		LPI		LSI		AI	
	2013	2022	2013	2022	2013	2022	2013	2022	2013	2022	2013	2022
LCZ1	0.35	0.30	57.00	51.00	92461170.36	82668071.48	0.03	0.05	8.05	7.22	13.55	15.15
LCZ2	0.76	0.89	109.00	120.00	176811711.75	194513109.37	0.03	0.04	11.75	12.37	13.51	16.42
LCZ3	1.30	0.66	233.00	124.00	377955310.43	200996879.69	0.03	0.04	16.33	11.62	8.61	7.38
LCZ4	3.42	4.43	415.00	535.00	673182205.28	867204279.29	0.22	0.33	23.10	26.31	19.85	19.89
LCZ5	1.76	2.99	296.00	463.00	480149235.57	750496413.66	0.06	0.11	18.76	23.55	9.90	12.86
LCZ6	16.83	13.34	1130.00	1096.00	1833002149.32	1776553065.61	1.14	0.54	45.66	43.26	29.48	24.85
LCZ8	3.17	3.06	357.00	318.00	579098909.12	515459739.84	0.18	0.32	21.09	20.73	25.40	25.53
LCZ9	6.15	9.77	836.00	1015.00	1356097165.34	1645256716.78	0.17	0.27	32.96	38.33	15.61	21.78
LCZ10	0.62	0.68	84.00	95.00	136258566.85	153989544.92	0.13	0.16	9.88	10.42	21.00	20.97
LCZA	16.20	13.34	317.00	336.00	514213877.29	544636706.24	8.05	4.10	26.75	26.99	58.40	53.78
LCZB	3.54	2.74	591.00	487.00	958676345.35	789399035.54	0.04	0.03	25.93	23.48	11.16	9.94
LCZC	0.82	1.03	178.00	214.00	288738391.66	346881711.72	0.03	0.02	13.55	15.28	2.93	3.59
LCZD	34.16	33.75	584.00	654.00	947321464.78	1060096446.08	11.55	6.71	38.78	40.08	58.28	56.59
LCZE	0.90	0.82	158.00	163.00	256295875.75	264213640.23	0.05	0.03	13.27	13.14	11.54	6.63
LCZF	0.84	2.95	184.00	410.00	298471146.44	664586457.02	0.01	0.08	14.00	22.78	2.08	16.00
LCZG	9.19	9.26	215.00	206.00	348757046.11	333914171.09	2.06	2.12	19.00	18.60	61.03	62.23
